# Transcriptome analysis of the compatible interaction of tomato with *Verticillium dahliae* using RNA-sequencing

**DOI:** 10.3389/fpls.2015.00428

**Published:** 2015-06-08

**Authors:** Guangxuan Tan, Kun Liu, Jingmin Kang, Kedong Xu, Yi Zhang, Lizong Hu, Ju Zhang, Chengwei Li

**Affiliations:** Key Laboratory of Plant Genetics and Molecular Breeding, Zhoukou Normal UniversityZhoukou, China

**Keywords:** tomato, *Verticillium dahliae*, compatible interaction, RNA sequencing, transcriptome analysis

## Abstract

Tomato *Verticillium* wilt is a soil-borne vascular disease caused by the necrotrophic fungus *Verticillium dahliae*. Although some understanding of plant defense mechanisms against *V. dahliae* infection has been gained for incompatible interactions, including identification of inducible resistant genes and defense signaling pathways, the genes and signaling pathways involved in the compatible interaction remain unclear. To investigate the molecular basis of the compatible interaction between tomato and *V. dahliae*, transcriptomes of *V. dahliae* infected tomatoes were compared to those of a control group. A total of approximately 25 million high-quality reads were generated by means of the RNA sequencing (RNA-seq) method. The sequence reads were aligned to the tomato reference genome and analyzed to measure gene expression levels, and to identify alternative splicing events. Comparative analysis between the two samples revealed 1,953 significantly differentially expressed genes (DEGs), including 1,281 up-regulated and 672 down-regulated genes. The RNA-Seq output was confirmed using RT-qPCR for 10 selected genes. The Nr, Swiss-Prot, Gene Ontology (GO), Clusters of Orthologous Groups (COG), and Kyoto Encyclopedia of Genes and Genomes (KEGG) databases were used to annotate DEG functions. Of the 1,953 DEGs identified, 1,953, 1,579, 1,739, 862, and 380 were assigned by Nr, Swiss-Prot, GO, COG, and KEGG, respectively. The important functional groups identified via GO and COG enrichment were those responsible for fundamental biological regulation, secondary metabolism, and signal transduction. Of DEGs assigned to 87 KEGG pathways, most were associated with phenylpropanoid metabolism and plant–pathogen interaction pathways. Most of the DEGs involved in these two pathways were up-regulated, and may be involved in regulating the tomato-*V. dahliae* compatible interaction. The results will help to identify key susceptible genes and contribute to a better understanding of the mechanisms of tomato susceptible response to *V. dahliae*.

## Introduction

*Verticillium* wilt is a serious vascular disease caused by the soil-borne fungus *Verticillium dahliae*, which is able to infect more than 200 plant species, including numerous economically important food crops (Klosterman et al., 2009). The infection of plants results from penetration of young roots by *V. dahliae* via wounds or cracks that occur at the sites of lateral roots (Fradin and Thomma, 2006; Klosterman et al., 2009). Once the fungus enters the xylem vessels, it rapidly multiplies and spreads along the xylem vessels to above-ground parts of the infected plant. In tomato plants, first the tips and edges of the lower leaves turn yellow, and V-shaped lesions form. Symptoms include stunted growth and extensive defoliation, which can be severe enough to lead to death of the plant (Fradin and Thomma, 2006). Plants have evolved an intricate and multilayered defense system to combat this infection, including hypersensitive responses and phenotypes resistant to infection. Plant responses to pathogens are usually classified as either host resistant (incompatible interaction) or host susceptible (compatible interaction) depending on the speed and extent of the visible reaction to infection and the ability of plant to limit pathogen growth. In general, following infection plant responses consist of three steps: the pathogen recognition, signal transduction, and the defense response itself, which involves many genes expressing defense-related proteins, which regulate complex signaling pathways (Jones and Dangl, 2006).

Tomato (*Solanum lycopersicum*) is one of the most economically important crops throughout the world. *Verticillium* wilt is a very important fungal disease of tomatoes and causes severe reductions in yield and quality in many parts of the world (Matta and Garibadli, 1997). The fungus develops extremely persistent resting structures known as microsclerotia that are capable of surviving in soil for many years. Chemical fumigation, the only effective control measure, is costly and has harmful environmental effects (Rowe et al., 1987; Fradin and Thomma, 2006). Efforts to develop varieties resistant to *V. dahliae* are frustrated by the emergence of new pathogen strains, which overcome the resistance. Therefore, the development of novel methods is necessary to control the disease, which demands a better understanding of the molecular mechanisms of interaction between tomato plants and the pathogen. To achieve the goal, it is of fundamental importance to uncover changes in tomato gene expression following infection, which will help to exploit both key genes in resistant and susceptible responses to *V. dahliae*. Besides the employment of multiple resistance genes in resistance breeding, knocking out (down) key susceptible genes could be an alternative method to develop cultivars with durable resistance.

Previous studies have used plant transcript profiling to identify differentially expressed genes (DEGs) in plant incompatible and compatible interactions to pathogens (Balaji et al., 2008; Ishihara et al., 2012; Du et al., 2014). In the compatible interaction between tomato and *Clavibacter michiganensis* ssp. *michiganensis*, the overwhelming majority of DEGs are induced, and it has been suggested that cell wall strengthening, oxidative burst, defense-related hormones and signaling, transcription factors and many pathogenesis related (PR) proteins have roles in basal defense (Balaji et al., 2008). Using analysis of DEGs in resistant and susceptible tomato lines during bacterial spot race T3 infection, Du et al. (2014) found similarities in the defensive mechanisms activated in the two tomato lines. In recent years, changes in gene expression induced by *V. dahliae* attack have been studied using transcriptional profiling in a number of plant-*V. dahliae* interactions (Gayoso et al., 2010; Xu et al., 2011; Sun et al., 2013). Expressions of various genes were increased or decreased in cotton resistance responses to *V. dahliae* (Xu et al., 2011; Zhang et al., 2013). It was also found that DEGs involved in lignin metabolism pathway played an important role in cotton resistance responses to *V. dahliae* (Xu et al., 2011). Using an RNA-seq approach, DEGs were identified in incompatible interactions between cotton and *V. dahliae*, and these were principally associated with cell wall, lipids, secondary metabolism, etc., (Sun et al., 2013). Microarray analysis showed that in both susceptible and tolerant interactions between tomato and *V. dahliae*, increased expressions of PR proteins were observed, but genes that were associated with foliar necrosis and cell death in the susceptible interaction appeared to be suppressed in the tolerant interaction (Robb et al., 2007). The identified induced DEGs were implicated in pathogen recognition, reactive oxygen species generation, phenylpropanoid metabolism, and phytohormone signaling. As a result, plants infected by the pathogen not only express a series of primary defense related genes but also activate phytohormone signal transduction and produce secondary metabolites such as phenylpropanoids.

Phenylalanine ammonia-lyase (PAL) is a key enzyme in phenylpropanoid metabolism, regulating lignin accumulation and the formation of defensive structures, along with and the synthesis of phenols which act as chemical defenses. Compatible and incompatible interactions between tomato and the pathogen both alter the timing of the onset of H_2_O_2_ production, Peroxidase activity, and PAL activity, but the response of susceptible tomato is slower and milder than that of resistant one (Gayoso et al., 2010). A similar result was also observed in cotton. Susceptible cotton strains showed obviously slower expression of lignin synthesis-related genes and PAL enzyme activity than those of resistant plants challenged by *V. dahliae* (Xu et al., 2011). In the compatible interaction between chickpea and the necrotrophic fungus *Ascochyta rabiei*, the up-regulated expression of PAL genes within 24 h of inoculation was verified by real-time PCR results (Jaiswal et al., 2012). In addition to gene changes related to the phenylpropanoid pathway in secondary metabolism, the expression of several phytohormone regulation molecules differs according to the type of pathogen attack. Plant defense response signals may be amplified through the generation of secondary signal molecules, such as salicylic acid (SA), ethylene (ET), and jasmonic acid (JA), which play an important role in defense signaling networks. In general, SA-dependent responses are involved in resistance to biotrophic or hemibiotrophic pathogens, whereas JA- and ET-mediated signaling pathways are activated in response to necrotrophic pathogens (Glazebrook, 2005). In *Arabidopsis* plants, infection with the biotrophic pathogen *Pseudomonas syringae*, which induces SA-mediated defense but suppresses the JA signaling pathway, rendered plants more susceptible to the necrotrophic pathogen *Alternaria brassicicola* (Spoel et al., 2007). A study in which tomato plants were challenged by the necrotrophic pathogen *A. alternata* f. sp. *lycopersici* suggests that the JA signaling pathway, which is dependent on the JAI1 receptor, is implicated in tomato susceptibility (Jia et al., 2013). In the compatible *Ascochyta*-chickpea interaction, most of the induced genes were transcriptionally up-regulated within 24 h of inoculation, these were involved in signaling and metabolic regulation, and included genes involved in the JA response (Jaiswal et al., 2012). These results suggest that the hormone pathways involved in plant defensive responses usually operate through complex networks of regulatory interactions.

Differential gene expression analyses have been carried out comparing transcription profiles from incompatible and compatible plant-*V. dahlia* interactions, and the functions of defense-related transcripts have been analyzed in detail (van Esse et al., 2009; Xu et al., 2011; Sun et al., 2013; Zhang et al., 2013). However, few studies have focused on differential expression profiles in compatible plant-*V. dahlia* interactions with the aim of identifying key genes in susceptible responses. In order to identify key functional genes in susceptible responses and understand the molecular basis of compatible interactions we used next-generation high-throughput RNA-seq to monitor and compare DEGs in tomato roots inoculated with *V. dahlia* with those in a control group over 2 days. The results revealed the transcriptome of tomato roots infected by *V. dahliae* and a large number of DEGs, and have the potential to assist in the development of new disease control strategies. The analyses of DEGs were focused on functional classification and the discovery of novel genes, particularly those involved in plant–pathogen interactions and secondary metabolic pathways.

## Materials and Methods

### Culturing of *V. dahliae*

The highly aggressive defoliating *V. dahliae* fungus isolate Vd080 (Li et al., 2014), which was kindly provided by Institute of Cotton Research, Chinese Academy of Agricultural Sciences, was cultured on potato dextrose agar (PDA) for 7 days at 25°C (Zhou et al., 2012). To obtain conidia the cultured isolate was then incubated in Czapek liquid medium for 5 days at 25°C. The spore suspension obtained was diluted to approximately 1 × 10^7^ spores per ml with sterile distilled water prior to inoculation.

### Plant Material and Inoculation Method

Seeds of the *Verticillium* susceptible tomato cultivar, Micro-Tom, were disinfected with 70% ethanol for 30 s and subsequently with 2.5% (v/v) sodium hypochlorite for 8–10 min. After each disinfection step, the seeds were washed with sterile water five times. The seeds were sown in culture bottles containing MS medium solidified with 0.8% plant agar. The seeds were pre-germinated at 4°C in the dark for 3 days, and subsequently transferred to a growth chamber at 25°C with a cycle of 16 h light (photosynthetic photon flux density 120 μmol⋅m^-2^⋅s^-1^) and 8 h dark. The roots of Micro-Tom seedlings with two fully expanded true leaves were inoculated with *Verticillium*. One milliliter of the *V. dahliae* spore suspension was directly pipetted onto the MS medium surface of each culture bottle. Control seedlings were similarly treated, but 1 ml of sterile water was substituted for the 1 ml of *V. dahliae* spore suspension. Each treatment was independently repeated five times in five individual culture bottles, and each bottle contained five seedlings. After inoculation, all culture bottles with seedlings were kept in the growth chamber at 25°C with a cycle of 16 h light (photosynthetic photon flux density 120 μmol⋅m^-2^⋅s^-1^) and 8 h dark.

### Determination of the Time-Point for Harvesting Samples

In order to mine tomato early responsive genes during compatible interaction with *V. dahliae*, the spatial and temporal expressions of genes require defining time points for collecting plant materials after inoculation. The early responsive genes of chickpea, involved in signaling and regulation of metabolic changes, were induced by fungus *A. rabiei* during compatible interaction 1 day post-inoculation (dpi; Jaiswal et al., 2012). The defense-related genes of susceptible tomato roots were elicited at 2 dpi in compatible interaction of tomato with *V. dahliae* (Gayoso et al., 2010). To observe Micro-Tom tomato response to *V. dahliae*, we performed the stem section cultivation from different time points after inoculation. Based on the protocol of fungal recovery assay described by Fradin et al. (2009), which minor modification was made, the stem sections of Micro-Tom tomatoes were cut at time points of 1, 2, 3, 4, 5, 6, and 7 dpi, respectively, and incubated on PDA at 25°C. *V. dahliae* mycelia were observed on the cultured stem sections collected from Micro-Tom tomatoes of 6 and 7 dpi, while no mycelium was observed on those of 1, 2, 3, 4, 5 dpi. It indicated the cotton roots of 2 dpi could be suitable for mining genes involved in the compatible interaction of cotton and *V. dahliae* based on the disease progress estimated by the length of stem sections to root tips of 6 and 7 dpi, which is coincided with the related previous reports (Gayoso et al., 2010; Sun et al., 2013). Therefore, the roots of 2 dpi for both pathogen-infected and control seedlings were sampled for the following RNA-Seq analysis. The roots harvested from five time repetitions of each individual treatment were mixed together and frozen immediately in liquid nitrogen for later use.

### RNA Extraction, Library Construction, and RNA-Seq

Total RNA was extracted from each root sample by using Trizol Reagent (Invitrogen, Life Technologies, Carlsbad, CA, USA) following the manufacturer’s protocol. Purified poly (A) + mRNA was extracted from the total RNA sample using Oligo(dT) magnetic beads. The mRNA was sheared into short fragments by adding a fragmentation buffer. First-strand cDNA was synthesized from these short poly (A) + mRNA fragments by adding random primers and SuperScript II. Buffer, dNTPs, DNA polymerase I, and RNaseH were then added to generate second-strand cDNA. The double-stranded cDNA was end-repaired by adding T4 DNA polymerase, Klenow Enzyme, and T4 polynucleotide kinase. This was followed by a single ‘A’ base addition using Klenow 3–5′ exo-polymerase, then sequencing adapters were ligated to the fragments using DNA ligase. For high-throughput sequencing, the cDNA fragments (PE200) were then separated by agarose gel electrophoresis and collected as sequencing templates. Finally, the cDNA library was constructed and sequenced on the Illumina HiSeq^TM^ 2000 platform.

### Alternative Splicing

To identify potential splicing sites that may contain information about exon boundaries and combinations in a transcript, paired-end reads were aligned to the *S. lycopersicum* reference genome (Zouine et al., 2012) using TopHat software set at the default parameters (Trapnell et al., 2009). According to all the junction sites of the same gene, we predicted types of alternative splicing (AS) events including exon skipping (ES), intron retention (IR), alternative 3′ splice site (A3SS), alternative 5′ splice site (A5SS), alternative first exon (AFE), and alternative last exon (ALE).

### Sequence Data and Differentially Expressed Gene Analysis

The raw reads were filtered, discarding sequences of adapters, reads with ambiguous bases ‘*N*’ and reads with more than 20% *Q* < 30 bases. All sequences smaller than 60 bp in length were also discarded following Meyer et al. (2009). The cleaned reads were aligned to the tomato genome (Zouine et al., 2012) using the spliced read mapper TopHat (Trapnell et al., 2012) version 2.0^1^. Transcript abundance and differential gene expression were calculated with the program Cuﬄinks (Trapnell et al., 2012)^2^. To compare gene expression levels between the two libraries, the relative transcript level of each expressed gene was calculated and normalized to the reads per kilobase of exon model per million mapped reads (RPKM) values (Mortazavi et al., 2008). Significant differences in gene expression were detected using the General Chi-squared test integrated in IDEG6 software^3^ (Romualdi et al., 2003). The *P* value threshold was determined by the false discovery rate (FDR) to account for multiple tests of significance. In this study, a FDR threshold ≤ 0.01 and Fold change ≥ 2 were adopted to judge the significance of the gene expression differences (i.e., the RPKM value of the gene in one sample was at least two times that of the gene in the other sample; Benjamini and Yekutieli, 2001).

To determine the functional annotation of DEGs, a BLAST (Basic Local Alignment Search Tool) alignment was performed by searching the Non-redundant (Nr), SwissProt, Kyoto Encyclopedia of Genes and Genomes (KEGG), and Clusters of Orthologous Groups (COG) protein databases with an *E*-value ≤ 1e-5. The best matches were selected to annotate the DEGs. Blast2go software^4^ was also used with an *E*-value ≤ 1e-5, to annotate the DEGs’ major Gene Ontology (GO) categories, including molecular functions, biological processes, and cellular components (Conesa and Götz, 2008).

### Real-Time Quantitative PCR (RT-qPCR) Analysis of the DEGs

Total RNA and cDNA syntheses were performed as described above to prepare and sequence the inoculated and control Micro-Tom tomato libraries. Primers of ten randomly selected DEGs and tomato actin gene as the control (Fradin et al., 2009) for RT-qPCR analysis were designed using DNAMAN6.0 software. RT-qPCR reactions were performed in 384-well plates using an ABI 7900HT Real Time PCR System (Applied Biosystems, Life Technologies, Carlsbad, CA, USA) and a SYBR^®^ *Premix Ex Taq*^TM^ (Tli RNaseH Plus), ROX plus (Takara Bio Inc., Shiga, Japan). The cycling conditions were as follows: 30 s denaturation at 95°C, followed by 40 cycles at 95°C for 5 s, and 60°C for 30 s. The relative expression levels were normalized and calibrated according to the 2^-ΔΔCT^ method (Livak and Schmittgen, 2001). Three biological replicates and three technical replicates were carried out for each of the selected genes.

## Results

### Sequence Analyzing and Aligning to the Reference Genome

To obtain a transcriptome profile of Micro-Tom tomato response to *V. dahliae*, two cDNA samples were extracted at 2 dpi from the roots of *V*. *dahliae*-inoculated (treatment) tomatoes and sterilized water-inoculated (control) tomatoes. The mRNA-seq libraries constructed for treatment or control tomatoes were sequenced using Illumina mRNA-Seq technology, generating more than 2 G of transcriptome data from each library. After stringent quality assessment and data cleaning, approximately 25 million (M) reads with 80% Q30 bases (those with a base quality greater than 30) were selected as high quality reads for further analysis. The high quality reads in this study were deposited in the NCBI SRA database (accession number: SRX1022130). An average ‘G + C’ content of above 40% (43.82, 45.68% for control and treatment libraries, respectively) was found. Of the selected reads, 89.15% from the control sample and 71.04% from the treatment sample were aligned onto the tomato reference genome and matched either unique or multiple genomic locations. The remaining 10.85 and 28.96% were unmapped on the tomato genome (**Table 1**).

**Table 1:** Summary of read numbers aligned onto the tomato reference genome.

Statistical content	Control		Treatment	
	Number	Percentage	Number	Percentage
Total reads	29,117,570	100%	21,893,066	100%
Mapped reads	25,957,067	89.15%	15,553,076	71.04%
Uniquely mapped reads	8,126,050	27.91%	4,588,929	20.96%
Multiple mapped reads	17,831,017	61.24%	10,964,147	50.08%
Unmapped reads	3,160,503	10.85%	5,739,990	28.96%

### Identification of Alternative Splicing Events

Alternative splicing produces diverse transcript variants from a single gene through the selective use of different splice sites, resulting in multiple protein products being generated from a single splicing gene (Nilsen and Graveley, 2010). Many plant genes undergo AS in response to pathogen attacks (Barbazuk et al., 2008). To investigate AS events in the compatible tomato- *V. dahliae* interaction, the sequence reads mapped to tomato genome regions were identified using computational analyses to find all theoretical splicing junctions. The following six types of AS events were present: alternative 3′ splice site (A3SS), alternative 5′ splice site (A5SS), ES, IR, AFE, and ALE (**Table 2**). Of the above six AS events, 7170 AS events from control samples and 5653 AS events from treatment samples were identified in 4833 and 4068 genes, respectively. IR was the most common type of AS, accounting for 30.8 and 32.8% of all AS events in control and treatment samples, respectively, while a rarer proportion of ALE events were detected in both control and treatment. Interestingly, the total number of AS events and the number of genes in which they occurred was lower in *V. dahliae* inoculated tomatoes than in control ones.

**Table 2:** Predicted Alternative splicing (AS) events in Micro-Tom tomato.

Alternative splicing type	Control	Percentage	Treatment	Percentage
Alternative 3′ splice site (A3SS)	1,173	16.4%	865	15.3%
Alternative 5′ splice site (A5SS)	670	9.3%	507	9.0%
Exon skipping (ES)	1,398	19.5%	1,091	19.3%
Intron retention (IR)	2,211	30.8%	1,853	32.8%
Alternative first Exon (AFE)	1,380	19.2%	1,071	18.9%
Alternative last Exon (ALE)	338	4.7%	266	4.7%
Total AS events	7170	100%	5653	100%
Loci having AS events	4833		4068	

### Functional Annotation and Classification of Differentially Expressed Genes

**FIGURE 1:**
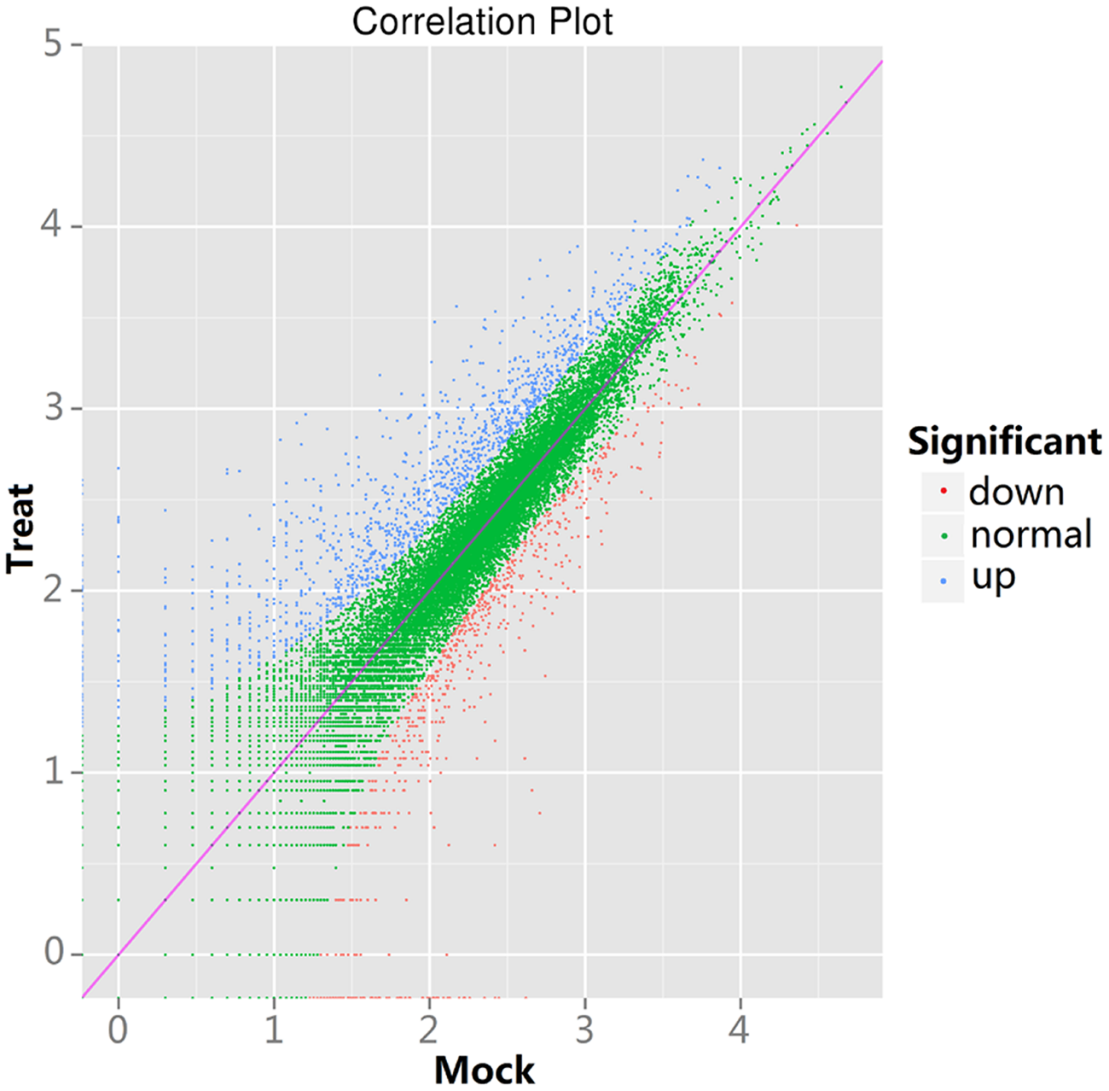
**Scatter plots of differentially expressed genes (DEGs) between control and treatment tomatoes**. The y-axis shows the expression level of treatment tomatoes and the x-axis is the expression level of control tomatoes.

To study the DEGs between the control and the treatment tomatoes, we employed a General Chi-squared test with FDR correction and a *P* value of 0.01 using IDEG6 software to identify twofold up-regulated and twofold down-regulated genes. Significantly different expression levels between the control and the treatment samples were found in 1,985 genes. Of those genes, 1,307 genes were up-regulated and 678 genes were down-regulated in the treatment sample (Supplementary Table S1). In **Figure 1**, the significantly up-regulated and down-regulated DEGs are indicated by blue and red dots, respectively.

To identify the main functional groups of the DEGs, we used a BLASTx search of the Nr, Swiss-Prot, KEGG, COG, and GO databases. This indicated that 1,953 DEGs (98.39%) had significant matches in the Nr database, with 1,579 (79.55%) in the Swiss-Prot database, and 380 (19.14%) in the KEGG database (**Table 3**). A total of 1,953 DEGs (98.39%) had annotation information in one or more of the Nr, Swiss-Prot, GO, KEGG, and COG databases (Supplementary Table S2).

**Table 3:** Functional annotation of the differentially expressed genes (DEGs) between control and treatment tomatoes.

Annotated databases	DEGs number	Percentage
Nr-annotation	1,953	98.39%
Swiss Prot-annotation	1,579	79.55%
Gene Ontology (GO)-annotation	1,739	87.71%
Kyoto Encyclopedia of Genes and Genomes (KEGG)-annotation	380	19.14%
Clusters of orthologous groups (COG)-annotation	862	43.43%
Total	1,953	98.39%

**FIGURE 2:**
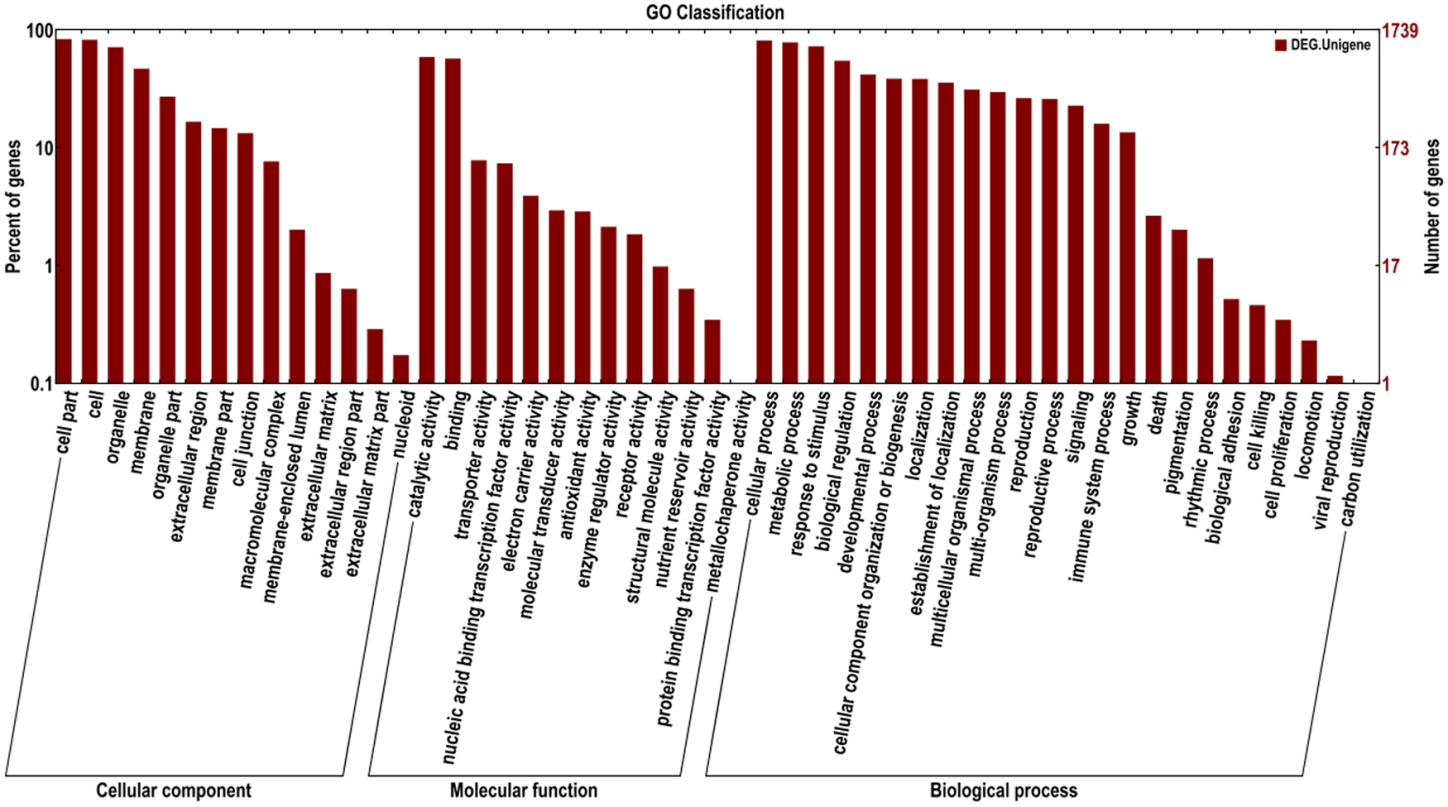
**Gene Ontology (GO) categories of DEGs between control and treatment tomatoes**.

The identified functional classes of the DEGs were subjected to GO enrichment analysis. According to the results of sequence alignments, 1,739 differential sequences were classified into 51 functional groups, belonging to three main categories: cellular components (1,563), molecular functions (1,430), and biological processes (1,620) (**Figure 2**). In the cellular component category, most DEGs were localized to cell part, cell and organelle and membrane. A few DEGs were localized to the extracellular matrix and nucleoid. In the molecular function category, a large number of DEGs were involved in catalytic activity and binding. In addition, a few DEGs belonged to two functional subclasses involved with protein binding transcription factor activity and metallochaperone activity. In the biological processes category, many DEGs were involved in cellular processes, metabolic processes, responses to stimuli, and biological regulation. Several DEGs were found to participate in biological adhesion, cell killing, cell proliferation, locomotion, viral reproduction, and carbon utilization. In total, these results indicate that most of the identified DEGs were responsible for fundamental processes associated with biological regulation and metabolism. KEGG pathway enrichment analysis was performed to categorize the biological functions of DEGs. A total of 290 DEGs were allocated to 87 KEGG pathways (Supplementary Table S3). The pathways involving the highest number of DEGs (29, 10.00%) were phenylalanine metabolism and phenylpropanoid biosynthesis, followed by plant hormone signal transduction (25; 8.62%), plant–pathogen interaction (24; 8.28%), cysteine and methionine metabolism (15; 5.17%), protein processing in endoplasmic reticulum (15; 5.17%), and oxidative phosphorylation pathways (15; 5.17%). These results suggest that phenylalanine metabolism and phenylpropanoid biosynthesis, signal transduction, and plant–pathogen interaction pathways were more involved in tomato susceptible response to *V. dahliae*, which is similar to those major pathways involved in plant and pathogen interactions in previous reports (Glazebrook, 2005; Robb et al., 2007; Spoel et al., 2007; Xu et al., 2011; Jaiswal et al., 2012; Sun et al., 2013; Zhang et al., 2013). Therefore, the DEGs involved in these pathways were considered as the candidates related to tomato susceptibility to *V. dahliae*.

According to the annotation from the COG database, 862 DEGs were classified into different COG classes. While 642 of 862 DEGs can be classified into 20 functional categories based on COG annotation, and the rest (220 DEGs) of 862 DEGs were without COG annotation. The DEGs of 20 functional categories were sequenced based on their number as follows, general function prediction only (104), posttranslational modification, protein turnover, chaperones (91), secondary metabolites biosynthesis, transport and catabolism (70), amino acid transport and metabolism (57), carbohydrate transport and metabolism (43), energy production and conversion (41), function unknown (38), inorganic ion transport and metabolism (34), lipid transport and metabolism (31), defense mechanisms (23), signal transduction mechanisms (21), cell wall/membrane/envelope biogenesis (19), transcription (16), translation, ribosomal structure and biogenesis (15), coenzyme transport and metabolism (14), intracellular trafficking, secretion, and vesicular transport (9), nucleotide transport and metabolism (7), replication, recombination and repair (5), cytoskeleton (3), RNA processing and modification (1). The DEGs involved in signal transduction mechanisms, secondary metabolites biosynthesis, transport and catabolism, and defense mechanisms are of interest because DEGs in these functional categories might participate in secondary metabolites and complex signaling pathways in response to the pathogen. In the 220 DEGs without COG annotation 94 DEGs belonging to COG class of RTKL were mainly homologous to receptor kinase genes based on the Nr annotation, suggesting that these DEGs could involve in the upstream of the tomato susceptible response pathway to *V. dahlia*. It indicated that more than 85% (80 DEGs) of the RTKL DEGs were up-regulated, implying that activated kinase genes were more in tomato susceptible response to *V. dahlia* than suppressed ones.

### Selection of the Core Candidates Related to Tomato Susceptibility to *V. dahliae*

It is important to select the candidate genes for further study after obtaining transcriptomic data. Based on the results of KEGG, COG, GO and Nr annotation, the first set of candidates related to tomato susceptibility to *V. dahliae* was selected from DEGs involved in three KEGG pathways (phenylalanine metabolism and phenylpropanoid biosynthesis, signal transduction, and plant–pathogen interaction) was selected as the candidates (Supplementary Table S4). The second set of candidates was selected from the rest of DEGs not included in KEGG pathway based on COG classification and the expression change levels of DEGs. For COG classes, which DEGs had higher expression change levels (up-regulated, log2FC > 3.5 or down-regulated, log2FC < -3.5), the up-regulated DEGs (log2FC > 3.5), and the down-regulated ones (log2FC < -3.5) were selected into the second set of DEGs; for COG classes, all of which DEGs had lower expression change levels (up-regulated, log2FC < 3.5 or down-regulated, log2FC > -3.5), the up-regulated DEG with the highest expression change level and down-regulated one with highest expression change level were selected for each COG class (Supplementary Table S4). The third set of candidates was selected from the rest of DEGs not included in KEGG pathway and COG classes based on the GO classification and the expression change levels of DEGs with the same standard as that for the candidate selection in COG classes. The fourth set of candidates was selected from the rest of DEGs not included in KEGG pathway, COG, and GO classes based on the expression change levels of DEGs with the same standard as that for the candidate selection in COG and GO classes (Supplementary Table S4). In the fourth set three new genes were included, for which no match was found in tomato cDNA database. In total 290 candidates were selected. These candidate DEGs will be further verified whether they are key genes involved in tomato susceptible responses to *V. dahliae.*

### Validation of Differentially Expressed Genes by RT-qPCR

**FIGURE 3:**
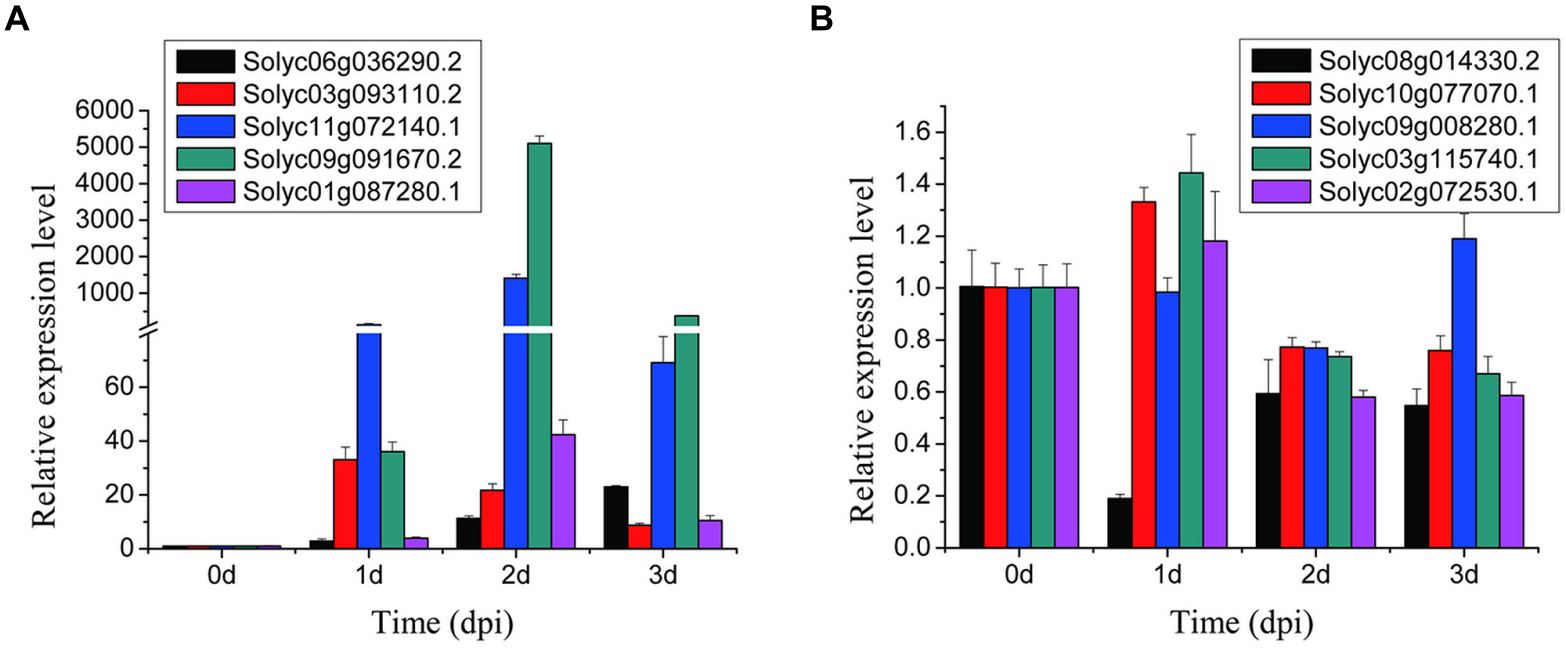
**Validation of the selected 10 DEGs in tomato Micro-Tom roots with RT-qPCR. (A,B)** represent five up-regulated and five down-regulated genes, respectively. SD with the average for three technical replicates are displayed.

In order to validate the gene expression data from RNA-seq, RT-qPCR of 10 randomly selected DEGs (five up-regulated and five down-regulated) was conducted with gene specific primers (Supplementary Table S5). Expression timing patterns of the investigated genes were analyzed in tomato root samples at four time-points (0, 1, 2, and 3 dpi). In each of three biological replicates, the expression patterns of the randomly selected DEGs according to RT-qPCR were in agreement with those obtained by the RNA-Seq at 2 dpi (**Figure 3**), suggesting that the RNA-seq data reflected the real expression patterns of the tomato genes in the compatible interaction.

## Discussion

In the biological process enrichment, the overwhelming majority of the DEGs were related to cellular processes, metabolic processes, responses to stimuli, and biological regulation. Similarly, in a UniGene analysis of cotton resistant response to *V. dahliae*, metabolic and cellular processes and responses to stimuli were also among the most highly represented groups associated with biological processes of (Zhang et al., 2013). It suggests that similar genes could be employed by both plant susceptible and resistant responses to *V. dahliae*. In the compatible interaction between tomato plants and the gram-positive bacterium *Clavibacter michiganensis* ssp. *michiganensis*, the basal defense responses of tomato are activated, and result in induction of a large number of defense genes, hormone involvement, oxygen metabolism, and protein degradation (Balaji et al., 2008). Our results show a similar activation of basal defense response related genes, suggesting that there may be an overlap among the susceptible responses of tomato to bacterial and fungal pathogens. In addition to the general function prediction, functional COG categories revealed that the identified DEGs were mainly involved in signal transduction mechanisms, transcription, secondary metabolism biosynthesis, transport and catabolism, similar to the results obtained in the biological process enrichment. It seems that, regardless of the functional annotation method applied the DEGs identified were mainly involved in metabolism and signal transduction. These results suggest that *V. dahliae* infection causes drastic metabolic changes in susceptible tomatoes leading to the accumulation of different proteins and secondary metabolites in roots, similar to the results about a previous study on tomato response to *V. dahliae* infection (Yadeta and Thomma, 2013).

To further understand the biological functions of the identified DEGs, a KEGG pathway enrichment analysis was carried out. Of the 87 pathways examined, the four pathways with the most associated DEGs (a total of 107) were phenylalanine metabolism, phenylpropanoid biosynthesis, plant hormone signal transduction, and plant–pathogen interaction. Among these DEGs associated with these four pathways, the overwhelming majority were up-regulated in roots of tomatoes that had received the pathogen inoculation treatment. In plant–pathogen interactions, the phenylpropanoid pathway plays a critical role in plant defense response to *V. dahliae* (Gayoso et al., 2010; Xu et al., 2011). It has also been reported that some genes involved in the phenylpropanoid pathway are induced during the compatible interaction of lettuce with the fungal pathogen *Botrytis cinerea* (de Cremer et al., 2013). Although this metabolic pathway was activated in both susceptible and resistant plants, the response of susceptible plants was slower and milder than that of resistant ones (Gayoso et al., 2010). The onset of the first key step in the pathway involves PAL which functions as a catalyst for phenylpropanoid metabolism. Gayoso et al. (2010) reported that PAL activity was increased in resistant tomato plants 2 hpi, while the induction of PAL activity in susceptible plants was not seen until 2 dpi. Of the six PAL genes with differing patterns of expression following *V. dahliae* inoculation, only PAL3 had increased expression 2 dpi in roots of susceptible plants, which coincided with an increase in PAL activity in the roots of inoculated susceptible plants. Our results also revealed that most of the DEGs involved in this pathway were up-regulated at 2 dpi. Intriguingly, two up-regulated DEGs were mapped to the node of phenylpropanoid biosynthesis (EC 4.3.1.24; K10775), which is associated with PAL1, PAL2, PAL3, and PAL4. One of the two genes, Solyc03g042560.1, corresponds with PAL1 in tomatoes, but the associated biological process is as yet unclear (Gayoso et al., 2010). The other gene, Solyc10g011930.1, is not designated as associated with any PAL in tomatoes in the databases of the National Center for Biotechnology Information^5^. Therefore, further research is necessary to elucidate the biological functions of these two PAL genes in susceptible tomatoes inoculated by *V. dahliae*.

Plant–pathogen interactions are complex processes that trigger a series of molecular responses at several expression levels. While resistant plants initiate responses in incompatible interactions, susceptible plants can also launch a series of basal defense responses in compatible interactions. Although they present similar expression profiles, defense gene induction in compatible interactions occurs later than that in incompatible interactions (Tao et al., 2003; Li et al., 2006; Balaji et al., 2008; Lara-Ávila et al., 2012). In the compatible chickpea–*Ascochyta* interaction, most of the genes analyzed were rapidly induced and transcriptionally up-regulated 1 dpi. Most of the up-regulated genes were related to plant–pathogen interactions (Jaiswal et al., 2012). Likewise, a study which compared the response of compatible potato inoculated with *Phytophthora infestans* to that of a control sample uncovered significant differential expression of many defense- and disease-responsive genes (Restrepo et al., 2005). In light of these facts, we selected one late time point (2 dpi), at which the expression profile of susceptible tomato roots was well revealed by RNA-seq. As mentioned before, more defense DEGs were induced and up-regulated in inoculated tomatoes in the control sample. Among these, in addition to those participating in the phenylpropanoid pathway, there were 24 DEGs assigned to nodes of plant–pathogen interaction pathways, of which 23 were up-regulated DEGs. In plant–pathogen interactions, plant hormone signaling transductions have been widely studied in different regulation pathways. In general, plants employ SA-dependent pathway against biotrophic pathogens, and adopt JA and ET signaling to combat necrotrophic pathogens (Glazebrook, 2005). JA signaling has been shown as key to basal resistance to necrotrophic fungi in tomatoes (AbuQamar et al., 2008; El-Oirdi et al., 2011). Several studies have confirmed that JA-ZIM domain (JAZ) is a negative regulator of JA signaling (Yan et al., 2007; Chung and Howe, 2009). Demianski et al. (2012) found that JAZ10 was one of the most highly induced genes among twelve JAZs induced in *Arabidopsis* by the bacterial pathogen *P. syringae* strain DC3000, and enhances susceptibility via a branch of the JA signaling pathway. Our results show that all four DEGs at the JAZ node were up-regulated in susceptible tomatoes. These DEGs were Solyc07g042170.2, Solyc12g009220.1, Solyc03g122190.2, and Solyc06g068930.1, which are assigned to the SlJAZ1, SlJAZ2, SlJAZ3, and SlJAZ8 tomato proteins, respectively (Sun et al., 2011). Whether these tomato JAZ proteins play a role in the regulation of JA signaling and promotion of tomato susceptibility to the necrotrophic fungus *V. dahliae* still needs further validation. Hence, verifying the biological functions of these genes is likely to help elucidate the molecular basis of susceptible responses to *V. dahliae*.

In this study about 66% of the total DEGs (1,953) were up-regulated and about 34% were down-regulated. For the DEGs involving in the upstream of the tomato susceptible response pathway to *V. dahlia*, the percentages of up-regulated genes were even higher. For example, for the DEGs belonging to COG class of RTKL, which were homologous to receptor kinase genes, more than 85% (80 DEGs) of the RTKL DEGs were up-regulated. It implied that susceptible response probably needs more genes to be activated than to be suppressed. However, the reason why different genes belonging to the same class showed different regulation patterns (up or down) in the tomato and *V. dahliae* interaction needs further study.

## Conclusion

Although the molecular mechanisms of plant defense responses to pathogens are increasingly being determined, few studies have shown that plants can become susceptible to disease via complex signaling pathways. This study provides valuable information concerning gene expression patterns of tomato roots susceptible to *V. dahliae*, which are believed to be molecular signaling mechanisms and may be part of a complex regulatory process. The present transcriptome analysis should promote further investigations into the detailed regulatory pathways regulating tomato susceptibility to *V. dahliae*, and contribute to a better understanding of the susceptible response of tomatoes to necrotrophic fungi. Future work should aim to further characterize the functions of the selected candidate DEGs involved in tomato-*V. dahliae* interactions (Supplementary Table S4), and elucidate the role of the genes involved in tomato susceptibility. This will help to determine the detailed regulatory mechanisms of plant diseases and develop new strategies for controlling tomato pathogens.

## Author Contributions

Conceived and designed the experiments: GT, CL. Performed the experiments: KL, JK. Analyzed the data: GT, KL JK, LH, CL. Contributed reagents/materials/analysis tools: JK, KX, YZ, JZ. Wrote the paper: GT, CL.

## Conflict of Interest Statement

The authors declare that the research was conducted in the absence of any commercial or financial relationships that could be construed as a potential conflict of interest.

## Footnotes

1 http://www.tophat.cbcb.umd.edu

2 http://cufflinks.cbcb.umd.edu/

3 http://telethon.bio.unipd.it/bioinfo/IDEG6/

4 http://www.blast2go.org

5 http://www.ncbi.nlm.nih.gov/
