# Supplementary material for: Transcriptome analysis of the compatible interaction of tomato with Verticillium dahliae using RNA-sequencing
Source: Front Plant Sci. 2015 Jun 8;6:428. doi: 10.3389/fpls.2015.00428 (PMC4458571; doi:10.3389/fpls.2015.00428)
Supplement: Supplementary file 5 [file Table_5.DOC]

**Table S5. Differentially expressed genes used for RT-qPCR verification**

| **Gene ID** | **Description** | **Regulation** | **Primer** |
| --- | --- | --- | --- |
| Solyc06g036290.2 | Heat shock protein 83-like isoform 1 | Up | **F:** 5’-AGACATCTACTACATCACT-3’  **R:** 5’-TTACCATCATATTCCTTCAG-3’ |
| Solyc03g093110.2 | Probable xyloglucan endotransglucosylase /hydrolase protein 23-like | Up | **F:** 5’-CAGTGCTAATGCTTGTATTC-3’  **R:** 5’-CTCTTGGCTTGTGTTATCTA-3’ |
| Solyc11g072140.1 | Wall-associated receptor kinase 3-like | Up | **F:** 5’-GATGGTAGTTGGCTGCGAT-3’  **R:** 5’-TGGGATTGAAGTCTGGCA-3’ |
| Solyc09g091670.2 | Pleiotropic drug resistance protein 1 | Up | **F:** 5’-GCCTACAGTCTTGCTCTCGT-3’  **R:** 5’-TTTCGTTGTTCTCACCCTC-3’ |
| Solyc01g087280.1 | Polygalacturonase-like | Up | **F:** 5’-AGCAGTGCGTGTGAGTAA-3’  **R:** 5’-TAAACCTCCTTCGTGACG-3’ |
| Solyc08g014330.2 | Copper methylamine oxidase-like | Down | **F:** 5’-CGATTGAGTTTCGTGGAG-3’  **R:** 5’-AGCATTCTTTCCGAGACC-3’ |
| Solyc10g077070.1 | Histone-lysine N-methyltransferase | Down | **F:** 5’-GTGCCTGGTTGGTTTACAC-3’  **R:** 5’-CAGAGGACACTATGCTAACAGC-3’ |
| Solyc09g008280.1 | S-adenosylmethionine synthase 3 | Down | **F:** 5’-GGGTATGCCACAGATGAAA-3’  **R:** 5’-ACAGTAACTTGGGTCTTGCC-3’ |
| Solyc03g115740.1 | Putative glycosyltransferase 5 | Down | **F:** 5’-GTTTCGGCAATCTCGCTT-3’  **R:** 5’-AAGGGTGAAGAAGTCATCCG-3’ |
| Solyc02g072530.1 | CBL-interacting serine/threonine-protein kinase 12-like isoform 1 | Down | **F:** 5’-GGTGTTTACCATAGGGACCT-3’  **R:** 5’-CCATCTTGCTTTATCTGCTC-3’ |
| Solyc04g011500 | Tomato actin (Reference gene) |  | **F:** 5’-CCATTCTCCGTCTTGACTTGG-3’  **R:** 5’-TCTTTCCTAATATCCACGTCAC-3’ |
